# Investigation of Direct Model Transferability Using Miniature Near-Infrared Spectrometers

**DOI:** 10.3390/molecules24101997

**Published:** 2019-05-24

**Authors:** Lan Sun, Chang Hsiung, Valton Smith

**Affiliations:** Viavi Solutions Inc., 1402 Mariner Way, Santa Rosa, CA 95407, USA; Chang.Hsiung@viavisolutions.com (C.H.); Valton.Smith@viavisolutions.com (V.S.)

**Keywords:** NIR, direct model transferability, MicroNIR™, SVM, hier-SVM, SIMCA, PLS-DA, TreeBagger, PLS, calibration transfer

## Abstract

Recent developments in compact near infrared (NIR) instruments, including both handheld and process instruments, have enabled easy and affordable deployment of multiple instruments for various field and online or inline applications. However, historically, instrument-to-instrument variations could prohibit success when applying calibration models developed on one instrument to additional instruments. Despite the usefulness of calibration transfer techniques, they are difficult to apply when a large number of instruments and/or a large number of classes are involved. Direct model transferability was investigated in this study using miniature near-infrared (MicroNIR™) spectrometers for both classification and quantification problems. For polymer classification, high cross-unit prediction success rates were achieved with both conventional chemometric algorithms and machine learning algorithms. For active pharmaceutical ingredient quantification, low cross-unit prediction errors were achieved with the most commonly used partial least squares (PLS) regression method. This direct model transferability is enabled by the robust design of the MicroNIR™ hardware and will make deployment of multiple spectrometers for various applications more manageable.

## 1. Introduction

In recent years, compact near infrared (NIR) instruments, including both handheld and process instruments, have attracted considerable attention and received wider adoption due to their cost-effectiveness, portability, ease of use, and flexibility in installation. These instruments have been used for various applications in different industries, such as the pharmaceutical industry, agriculture, the food industry, the chemical industry, and so on. [1,2,3,4,5] They enable point-of-use analysis that brings advanced laboratory analysis to the field [6,7] and online and inline analysis that permits continuous process monitoring [8,9]. Moreover, scalability of NIR solutions has become possible. It is common that users of compact NIR instruments would desire more than one instrument to be used for their applications. Sometimes a large number of instruments are deployed.

Intrinsically, NIR solutions require multivariate calibration models for most applications due to the complexity of the spectra resulting from vibrational overtones and combination bands. Usually a calibration data set is collected using an NIR instrument to develop a calibration model. However, when multiple instruments are deployed for the same application, it is too time and labor consuming to collect calibration sets and develop calibration models for these instruments individually. It is also very inconvenient to manage different calibration models for different instruments. Therefore, it is highly desirable that calibration development is performed only once, and that the calibration model can be used on all these instruments successfully. In practice, when multiple instruments are involved for a particular application, the calibration model is often developed on one instrument and then applied to the rest of the instruments, especially when a project starts with one instrument for a feasibility test and then multiple instruments are procured. When a large number of instruments are involved, a global model approach can be taken in which calibration data from at least two to three instruments are pooled to develop the calibration model, in order to minimize noncalibrated variations from the instruments [10]. For any of the cases, model transferability from one or multiple instruments to the others is critical. 

Historically, instrument-to-instrument variations could prohibit the success of the direct use of calibration models developed on one instrument with the other instruments. To avoid full recalibration, various calibration transfer methods have been developed to mathematically correct for instrument-to-instrument variations [10,11]. Common methods include direct standardization [12], piecewise direct standardization (PDS) [12,13,14], spectral space transformation [15], generalized least squares (GLS) [16], and so on. These methods have been extensively used to transfer quantitative calibration models [17,18,19,20], but very few studies were focused on the transfer of classification models [21,22]. Although these methods are very useful, they can only deal with calibration transfer from one instrument to another at a time and require transfer datasets to be collected from the same physical samples with both instruments. This is practical when there are only a few instruments involved. One instrument can be designated as the master instrument to develop the calibration model. Then data collected by the other instruments can be transformed into the master instrument’s approximate space via the respective pair of transfer datasets. Thus, the master calibration model can be used by the other instruments. Alternatively, the master calibration data can be transferred to the other target instruments and calibration models can be developed on these target instruments. However, in the new era of handheld and process NIR instrumentation, a large number of instruments (e.g., > 20) could be deployed for one application. It would be difficult to perform calibration transfer in this way, especially when these instruments are placed in different locations. Other calibration transfer methods have been developed without using the transfer datasets from both instruments [23,24,25]. But unlike the commonly used methods, these methods have not been extensively studied and made easily available to general NIR users. Moreover, calibration transfer of classification models typically requires transfer data to be collected from every class. When a large number of classes are included in the model, the efforts required would be close to rebuilding a library on the secondary instrument. This may explain why very few studies have been conducted on transfer of classification models. 

Considering all the advantages and potentials the handheld and process NIR instruments can offer and the challenges for calibration transfer when a large number of instruments and/or a large number of classes are involved, it is intriguing to understand if advances in instrumentation and modeling methods could make direct use of the master calibration model acceptable. However, to the best of our knowledge, little research has been done in this area. 

The authors have demonstrated in the past that the use of miniature near-infrared (MicroNIR™) spectrometers with the aid of support vector machine (SVM) modeling can achieve very good direct transferability of models with a large number of classes for pharmaceutical raw material identification [26]. In the current study, using MicroNIR™ spectrometers, direct model transferability was investigated for polymer classification. Five classification methods were tested, including two conventional chemometric algorithms, partial least squares discriminant analysis (PLS-DA) [27] and soft independent modeling of class analogy (SIMCA) [28], and three machine learning algorithms that are burgeoning in chemometrics, bootstrap-aggregated (bagged) decision trees (TreeBagger) [29], support vector machine (SVM) [30,31] and hierarchical SVM (hier-SVM) [26]. High cross-unit prediction success rates were achieved. Direct transferability of partial least squares (PLS) regression models was also investigated to quantify active pharmaceutical ingredients (API). Low cross-unit prediction errors were obtained.

## 2. Results

### 2.1. Classification of Polymers

Polymers are encountered in everyday life and are of interest for many applications. In this study polymer classification was used as an example to investigate direct model transferability. Resin kits containing 46 materials representing the most important plastic resin used in industry today were used. Each material was treated as one class. Three resin kits were used to show prediction performance on different physical samples of the same material. The samples were measured by three randomly chosen MicroNIR™ OnSite spectrometers (labeled as Unit 1, Unit 2 and Unit 3).

#### 2.1.1. Spectra of the Resin Samples

**Figure 1:**
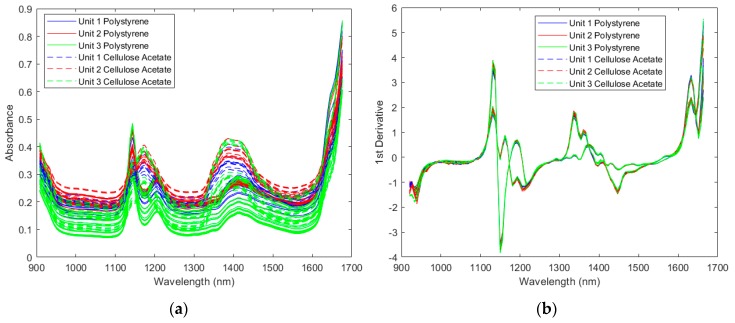
Spectra of example polymer samples by three instruments: (**a**) raw spectra; (**b**) preprocessed spectra by Savitzky-Golay 1st derivative (5 smoothing points and 3rd polynomial order) and standard normal variate (SNV).

Spectra collected by the three spectrometers were compared in Figure 1. For clarity, example spectra of two samples were presented. The same observations were obtained for the other samples. The raw spectra in Figure 1a only show baseline shifts between measurements using different spectrometers for the same sample. These shifts were mainly due to different measurement locations, since these resin samples are injection molded and are not uniform in thickness and molecular orientation. In fact, baseline shifts were also observed when using the same spectrometer to measure different locations of the same sample. These shifts can be corrected by spectral preprocessing, and the preprocessed spectra from the same sample collected by different spectrometers were very similar as shown in Figure 1b.

#### 2.1.2. Direct Model Transferability of the Classification Models

The performance of the polymer classification models was evaluated at four levels, the same-unit-same-kit performance, the same-unit-cross-kit performance, the cross-unit-same-kit performance, and the cross-unit-cross-kit performance. To account for the most variation in sample shape and thickness, each resin sample was scanned in five specified locations. In addition, at each position the sample was scanned in two orientations with respect to the MicroNIR™ lamps to account for any directionality in the structure of the molding. For each position and orientation, three replicate scans were acquired, totaling thirty scans per sample, per spectrometer. Prediction was performed for every spectrum in the validation set. For the same-unit-same-kit performance, the models built with data collected from four locations on each sample in one resin kit by one spectrometer were used to predict data collected from the other location on each sample in the same resin kit by the same spectrometer. The total number of predictions was 276 for all 46 materials for each case. For the same-unit-cross-kit performance, the models built with all the data collected from one resin kit by one spectrometer were used to predict all the data collected from a different resin kit by the same spectrometer. The total number of predictions was 1380 for all 46 materials for each case. For the cross-unit-same-kit performance, the models built with all the data collected from one resin kit by one spectrometer were used to predict all the data collected from the same resin kit by a different spectrometer. The total number of predictions was 1380 for all 46 materials for each case. For the cross-unit-cross-kit performance, the models built with all the data collected from one resin kit by one spectrometer were used to predict all the data collected from a different resin kit by a different spectrometer. The total number of predictions was 1380 for all 46 materials for each case. Five different classification algorithms were used to build the models, which were PLS-DA, SIMCA, TreeBagger, SVM and hier-SVM. 

The prediction performance was evaluated in terms of prediction success rates and the number of missed predictions. The representing results were summarized in Table 1 and Table 2, respectively. The prediction success rates were calculated by dividing the number of correct predictions with the number of total predictions. The number of missed predictions is presented to make the difference clearer, since with a large number of total predictions a small difference in prediction success rate would mean a conceivable difference in the number of missed predictions. It should be noted that in a few cases the total number of predictions was not exactly 276 or 1380, because extra spectra were collected unintentionally during experiments and no spectra were excluded from analysis. To make the comparison consistent, in these tables all the models were developed using data from Kit 1 for different spectrometers. The prediction data were collected using different resin kits and different spectrometers for the four levels of performance. 

The same-unit-same-kit cases were control cases and presented as the diagonal elements for each algorithm in the left three columns of the tables. As expected, 100% prediction success rates and 0 missed predictions were obtained for all algorithms except for one PLS-DA case (Unit 1 K1 for modeling and testing) where there was only 1 missed prediction. The same-unit-cross-kit cases showed the true prediction performance of the models for each spectrometer, since independent testing samples were used. The results are presented as the diagonal elements for each algorithm in the right three columns of the tables. All the models showed very good same-unit-cross-kit predictions. Although SIMCA showed the best performance, the differences in performance were very small between algorithms. It should be noted that samples made of the same type of material but with different properties are included in the resin kits, indicating that the MicroNIR™ spectrometers have the resolution to resolve minor differences between these polymer materials. For the cross-kit cases, Kit 2 was used for Unit 1 and Unit 2, while Kit 3 was used for Unit 3, because at the time of data collection using Unit 3, Kit 2 was no longer available. Nonetheless, conclusions about the cross-kit performance were not impacted by this.

The direct model transferability was first demonstrated by the cross-unit-same-kit results, which are presented by the non-diagonal elements for each algorithm in the left three columns of the tables. Except the PLS-DA algorithm, all the other algorithms showed good performance. In general, the order of performance was Hier-SVM > SVM > SIMCA > TreeBagger >> PLS-DA. When the hier-SVM algorithm was used, the worst case only had 28 missed predictions out of 1380 predictions, and 1/3 of the cases showed perfect predictions.

The direct model transferability was further demonstrated by the most stringent cross-unit-cross-kit cases, which are often the real-world cases. The results are presented by the non-diagonal elements for each algorithm in the right three columns of the tables. Other than the PLS-DA algorithm, all the other algorithms showed good performance, but which was slightly worse than the cross-unit-same-kit results with some exceptions. In general, the order of performance was hier-SVM > SVM > TreeBagger ≈ SIMCA >> PLS-DA. 

Besides the representing results shown in these tables, all possible combinations of datasets were analyzed, including 6 same-unit-same-kit cases, 6 same-unit-cross-kit cases, 8 cross-unit-same-kit cases, and 16 cross-unit-cross-kit cases in total for each algorithm. The conclusions were similar to those presented above. For the most stringent cross-unit-cross-kit cases, the mean prediction success rates of all the cases were 98.15%, 97.00%, 96.74%, 95.83%, and 80.19% for hier-SVM, SVM, TreeBagger, SIMCA, and PLS-DA, respectively. The high prediction success rates for hier-SVM, SVM, TreeBagger and SIMCA indicate good direct model transferability for polymer classification with MicroNIR™ spectrometers. To achieve the best result, hier-SVM should be used. But the conventional SIMCA algorithm that is available to most NIR users is also sufficient. 

### 2.2. Quantification of Active Pharmaceutical Ingredients

Quantitative analysis of an active pharmaceutical ingredient is important in several different steps of a pharmaceutical production process and it was proved that NIR spectroscopy is a good alternative to other more time-consuming means of analysis [32]. As one of the process analytical technology (PAT) tools adopted by the pharmaceutical industry, compact NIR spectrometers can be installed for real-time process monitoring, enabling the quality by design (QbD) approach that is now accepted by most pharmaceutical manufacturers to improve manufacturing efficiency and quality [33,34]. In this context, multiple NIR spectrometers will be needed for the same application. It is important to understand the direct transferability of calibration models to determine APIs quantitatively.

To investigate this, a five-component pharmaceutical powder formulation including three APIs, acetylsalicylic acid (ASA), ascorbic acid (ASC), and caffeine (CAF), as well as two excipients, cellulose and starch, was used. A set of 48 samples was prepared by milling varying amounts of the three APIs in the concentration range of 13.77–26.43% *w*/*w* with equal amounts (40% *w*/*w*) of a 1:3 (*w*/*w*) mixture of cellulose and starch [4]. The set of samples was measured by three randomly chosen MicroNIR™ 1700ES spectrometers (labeled as Unit 1, Unit 2 and Unit 3). 

#### 2.2.1. Spectra of the Pharmaceutical Samples

**Figure 2:**
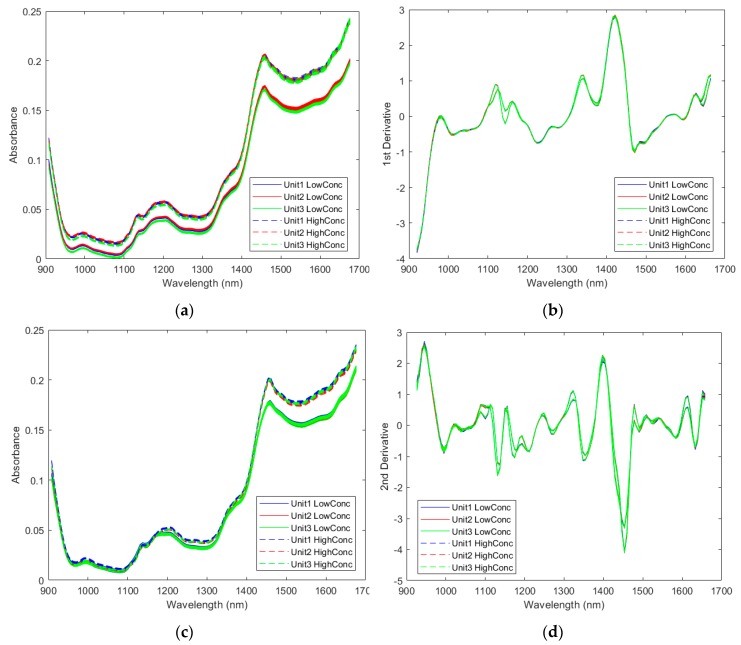

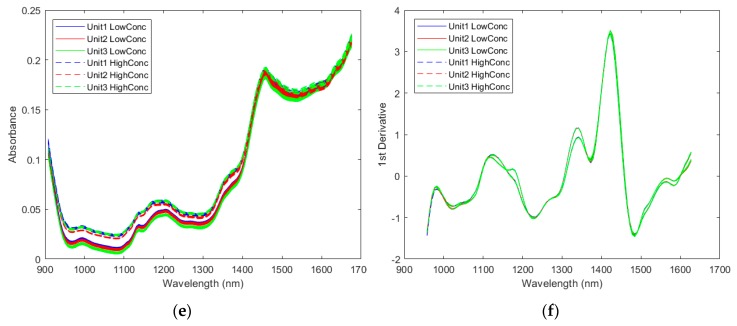
Spectra of samples with the highest and the lowest active pharmaceutical ingredient (API) concentrations measured by three instruments: (**a**) selected raw spectra based on the acetylsalicylic acid (ASA) concentration; (**b**) selected preprocessed spectra based on the ASA concentration by Savitzky-Golay 1st derivative (5 smoothing points and 2nd polynomial order) and SNV; (**c**) selected raw spectra based on the ascorbic acid (ASC) concentration; (**d**) selected preprocessed spectra based on the ASC concentration by Savitzky-Golay 2nd derivative (7 smoothing points and 3rd polynomial order) and SNV; (**e**) selected raw spectra based on the caffeine (CAF) concentration; (**f**) selected preprocessed spectra based on the CAF concentration by Savitzky-Golay 1st derivative (17 smoothing points and 3rd polynomial order) and SNV.

The spectra were first compared across the three instruments. Raw spectra of two samples with the lowest ASA concentration and the highest ASA concentration collected by all three instruments are shown in Figure 2a. Only slight baseline shifts can be seen between spectra collected by different instruments. The preprocessed spectra collected by different instruments became almost identical, as shown in Figure 2b. However, spectral differences between the high concentration sample and the low concentration can be clearly seen. Similar observations were obtained for the other two APIs, ASC (Figure 2c,d) and CAF (Figure 2e,f). It should be noted the optimized preprocessing steps were chosen to generate the preprocessed spectra for each API, respectively. 

#### 2.2.2. Direct Model Transferability of the Quantitative Models

To develop the quantitative calibration models, 38 out of the 48 samples were selected as the calibration samples via the Kennard-Stone algorithm [35], based on the respective API concentration, which was determined by the amount of API added to the powder sample. The remaining 10 samples were used as the validation samples. Twenty spectra were collected from each sample with every spectrometer. Thus, 760 spectra from the 38 calibration samples were used to build every model and 200 spectra from the 10 validation samples were used to validate each model. For each API, an individual model was developed on each instrument by partial least squares (PLS) regression. Different preprocessing procedures with different settings were tested and the optimal one was determined based on the cross-validation statistics using the calibration set. The same optimal preprocessing procedure was selected on all three instruments for the same API. The API models were developed using the corresponding preprocessed spectra.

The model performance was first evaluated in terms of normalized root mean square error of prediction (NRMSEP), which is root mean square error of prediction (RMSEP) normalized to the mean reference value of the validation set. NRMSEP was used to provide an estimate of how big the error was relative to the value measured. Since the mean reference value was the same for all the validation sets, it is equivalent to comparing RMSEP. Two types of prediction performance were examined, the same-unit performance and the cross-unit performance. Using a calibration model developed on one instrument, the same-unit performance was determined by predicting the validation set obtained with the same instrument, and the cross-unit performance was determined by predicting the validation set obtained with a different instrument. The cross-unit performance is the indicator of direct model transferability. The results were reported under the No Correction section in Table 3, Table 4 and Table 5 for ASA, ASC and CAF, respectively. The unit number in the row title represents which of the instruments was used to develop the calibration model, and the unit number in the column title represents which instrument was used to collect the validation data. Therefore, the NRMSEP values on the diagonal indicate the same-unit performance, while the other values indicate the cross-unit performance. The data show that cross-unit performance was close to the same-unit performance, all below 5%. 

In another independent study, the same samples were measured by a benchtop Bruker Vector 22/N FT-NIR spectrometer. The reported mean absolute bias based on 3 validation samples was 0.28, 0.62 and 0.11 for ASA, ASC and CAF, respectively [36]. In the current study, the mean absolute bias of the three same-unit cases based on 10 validation samples was 0.21, 0.35 and 0.22 for ASA, ASC and CAF, respectively. The mean absolute bias of the six cross-unit cases based on 10 validation samples was 0.14, 0.30 and 0.25, respectively. These results indicate that both the same-unit and the cross-unit MicroNIR™ performance is comparable with the benchtop instrument performance. However, it should be noted in the current study 38 samples were used for calibration and 10 samples were used for validation, while in the other study 45 samples were used for calibration and 3 samples were used for validation. 

**Figure 3:**
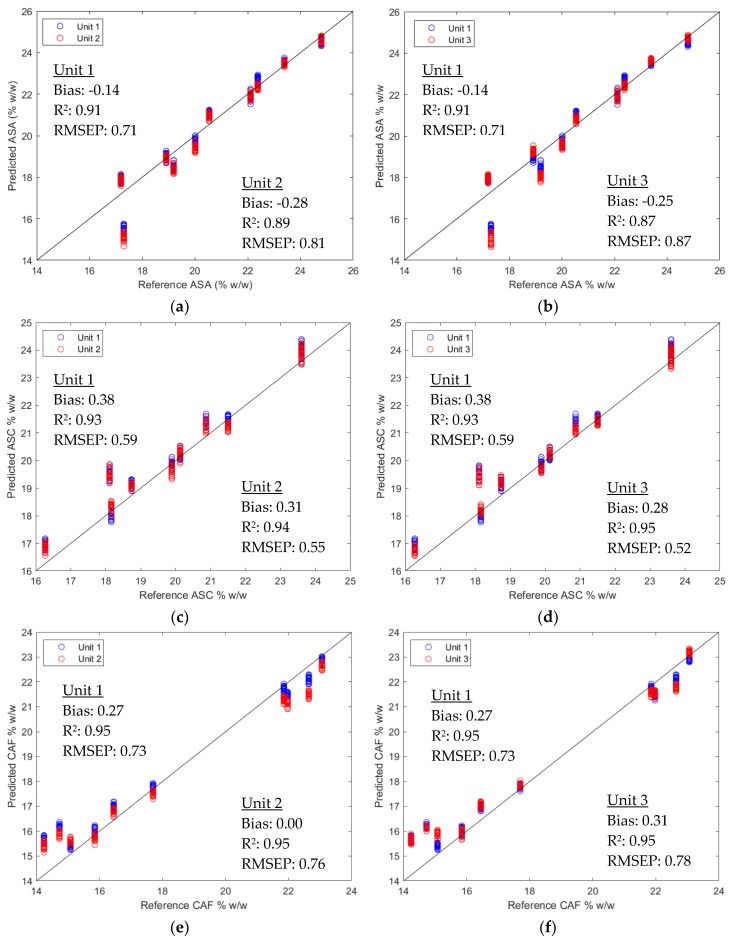
Predicted values versus reference values using models developed on Unit 1: (**a**) validation sets by Unit 1 and Unit 2 for ASA prediction; (**b**) validation sets by Unit 1 and Unit 3 for ASA prediction; (**c**) validation sets by Unit 1 and Unit 2 for ASC prediction; (**d**) validation sets by Unit 1 and Unit 3 for ASC prediction; (**e**) validation sets by Unit 1 and Unit 2 for CAF prediction; (**f**) validation sets by Unit 1 and Unit 3 for CAF prediction. The corresponding bias, R^2^ for prediction, and root mean square error for prediction (RMSEP) are presented in each plot.

The model performance was further examined by the predicted values of the validation set versus the reference values. Using calibration models developed on Unit 1, the same-unit predicted results and the cross-unit predicted results for ASA, ASC and CAF are shown in Figure 3. It can be seen that most of the predicted values stay close to the 45-degree lines, explaining the good model performance. Moreover, the cross-unit results (red circles) are very close to the same-unit results (blue circles), explaining the similar cross-unit performance to the same-unit performance.

**Figure 4:**
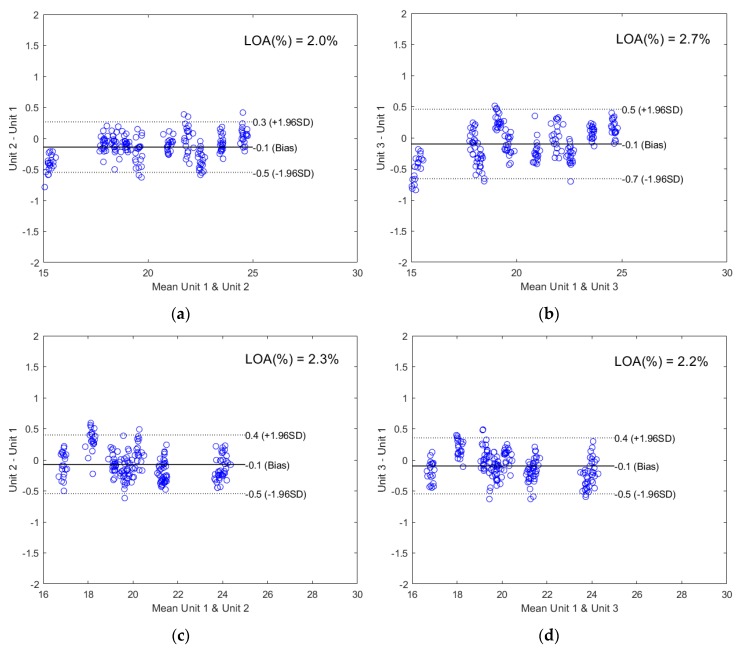

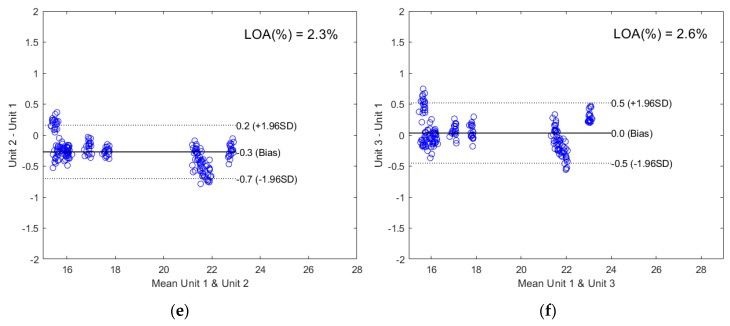
The Bland-Altman plots comparing the cross-unit prediction results and the same-unit prediction results using models developed on Unit 1: (**a**) validation sets by Unit 1 and Unit 2 for ASA prediction; (**b**) validation sets by Unit 1 and Unit 3 for ASA prediction; (**c**) validation sets by Unit 1 and Unit 2 for ASC prediction; (**d**) validation sets by Unit 1 and Unit 3 for ASC prediction; (**e**) validation sets by Unit 1 and Unit 2 for CAF prediction; (**f**) validation sets by Unit 1 and Unit 3 for CAF prediction.

The corresponding Bland-Altman plots were used to illustrate the agreement between the cross-unit prediction results and the same-unit prediction results in Figure 4. The Bland-Altman analysis is a well-accepted technique for method comparison in highly regulated clinical sciences [37] and shows good visual comparison between two instruments [11]. The x-axis shows the mean predicted value and the y-axis shows the difference between the cross-unit predicted value and the same-unit predicted value. The limits of agreement (LOA) were calculated by Equation (1):

(1) $$LOA=\bar{d}\pm 1.96\times SD$$

where $\bar{d}$ is the bias or the mean difference, and SD is the standard deviation of the differences. It can be seen from Figure 4 that with only a few exceptions, all data points stayed within the LOA, indicating that at a 95% confidence level, the cross-unit prediction results agreed well with the same-unit prediction results. LOA relative to the mean of the mean predicted values (x-axis) was below 3% for all three APIs. 

**Figure 5:**
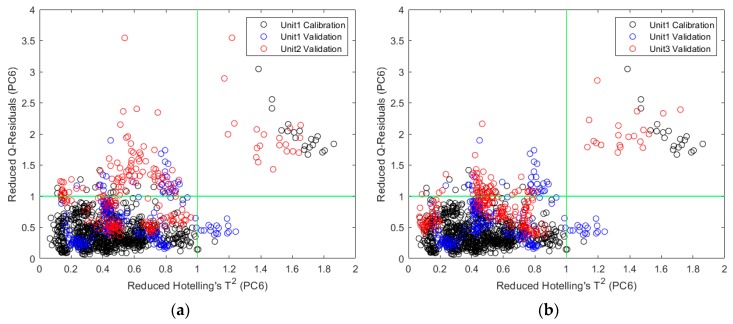

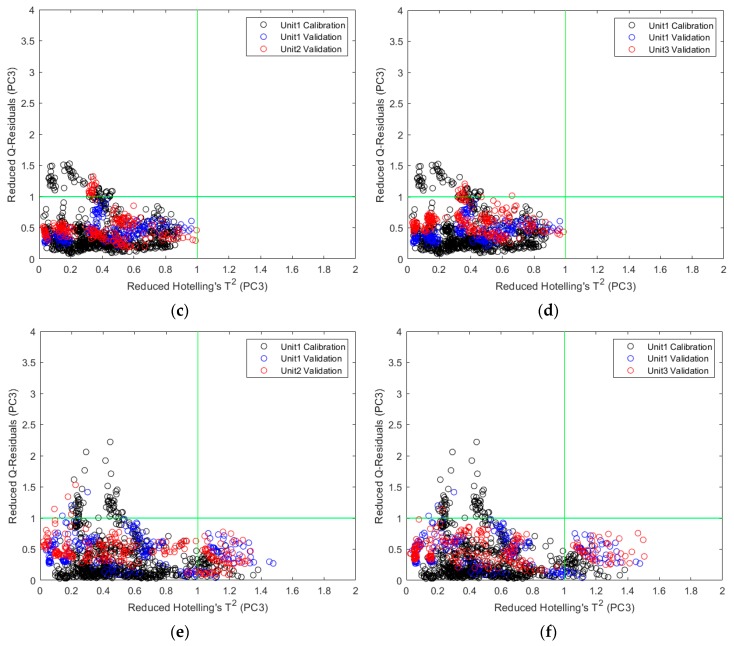
Reduced Q residuals versus reduced Hotelling’s T^2^ for models developed on Unit 1: (**a**) validation sets by Unit 1 and Unit 2 for ASA prediction; (**b**) validation sets by Unit 1 and Unit 3 for ASA prediction; (**c**) validation sets by Unit 1 and Unit 2 for ASC prediction; (**d**) validation sets by Unit 1 and Unit 3 for ASC prediction; (**e**) validation sets by Unit 1 and Unit 2 for CAF prediction; (**f**) validation sets by Unit 1 and Unit 3 for CAF prediction.

The corresponding reduced Hotelling’s T^2^ and reduced Q residuals are shown in Figure 5. The reduced statistics were calculated by normalizing Hotelling’s T^2^ and Q residuals to their respective 95% confidence limit. The black circles represent the calibration data, the blue circles represent the same-unit validation data, and the red circles represent the cross-unit validation data. It can be clearly seen that the cross-unit validation data stayed close to the same-unit validation data, further explaining the similar cross-unit performance to the same-unit performance. It was noticed that 20 calibration data points (from the same physical sample) and 20 cross-unit validation data points (from another physical sample) are in the high reduced Hotelling’s T^2^ and high reduced Q residuals quadrant for ASA (Figure 5a,b). These explained why the prediction results of one sample significantly deviated from the 45-degree lines in Figure 3a,b. However, to keep the analysis consistent with the other two APIs and data available in literature [4,36] for comparison, no sample was excluded from calibration or validation.

#### 2.2.3. Calibration Transfer

To check how direct model transfer compared with calibration transfer, three types of calibration transfer methods were tested. The first method was bias correction by standardizing the predicted values, which is probably the simplest method. The second method was PDS by mapping spectral responses of the slave instrument to the master instrument, which is probably the most commonly used method. The third method was GLS by removing the differences between instruments from both instruments. To perform the calibration transfer, 8 transfer samples were selected from the calibration samples with the Kennard-Stone algorithm. The calibration transfer results using Unit 1 as the master instrument were summarized in Table 3, Table 4 and Table 5 for ASA, ASC and CAF, respectively. It should be noted that different settings for PDS and GLS were tested. The results presented were obtained under the best settings based on RMSEP. By comparing these results with the corresponding same-unit and cross-unit results (Column 1 under No Correction), there was not a single method that could improve cross-unit results for all three APIs. Choosing the best method for individual API, only slight improvement (decrease of 0.3–0.9% in RMSEP%) of cross-unit performance was observed. Calibration transfer could sometimes damage the performance when a certain method was applied to a certain API. In addition, for ASC and CAF, the cross-unit performance was already close to or slightly better than the same-unit performance. For ASA, although the same-unit performance was better than the cross-unit performance using the calibration model on Unit 1 (Column 1 under No Correction in Table 3), it was similar to the cross-unit performance using calibration models on Unit 2 and Unit 3 (Row 1 under No Correction in Table 3). All these observations indicate that the instrument-to-instrument difference was small. Therefore, calibration transfer may not be necessary for this application.

## 3. Discussion

The good direct model transferability demonstrated in this study was enabled by the minimal instrument-to-instrument differences owing to the robust design of the MicroNIR™ hardware. The MicroNIR™ spectrometer utilizes a wedged linear variable filter (LVF) as the dispersive element on top of an InGaAs array detector, which results in an extremely compact and rugged spectral engine with no moving parts [4]. The operation of the on-board illumination allows for a steady output of optical power and an extended lamp-life. Thus, a very stable performance can be achieved without the need for realignment of hardware over time. In addition to the hardware design, the performance of every MicroNIR™ spectrometer is evaluated and calibrated at the production level. The accuracy of the MicroNIR™ wavelength calibration enables precise spectral alignments from instrument to instrument. The repeatability of the photometric response ensures the consistency of signal amplitude from instrument to instrument. The unit-specific temperature calibration stabilizes the MicroNIR™ response over the entire operating temperature range. In the Supplementary Material, the wavelength reference plots and the photometric response plots are shown for the MicroNIR™ OnSite units used for the polymer classification example (Figure S1) and the MicroNIR™ ES units used for the API quantification example (Figure S2), respectively. Very small instrument-to-instrument differences were observed. It should be noted that findings from the handheld MicroNIR™ OnSite and ES units could be extended to the MicroNIR™ PAT units for process monitoring, since the spectral engine and the calibration protocol at the production level are the same. 

In this study, both a classification example and a quantification example were investigated. For the quantification example, the good direct model transferability was demonstrated with the most commonly used regression method, PLS. For the classification example, the good direct model transferability was demonstrated with both the commonly used chemometric algorithm, SIMCA, and the machine learning algorithms, SVM, hier-SVM and TreeBagger. It should be noted the PLS-DA performance could be improved to about 90% prediction success rate by manually optimizing the number of PLS factors. The results presented in Table 1 and Table 2 were based on automatically selected PLS factors. This automatic selection procedure sometimes causes overfitting. However, since all the other algorithms were also using automatic model building, which may not always generate the best results, for a fair comparison no manual intervention was introduced to PLS-DA. In fact, even with the improved performance, PLS-DA still didn’t perform as well as the other algorithms for this specific application. Although the direct model transferability was good with conventional SIMCA, it can be further improved with the use of SVM algorithms. SVM has found increasing interest in chemometrics in recent years, since it is such a sound methodology, where geometric intuition, elegant mathematics, theoretical guarantees, and practical algorithms meet [38]. Among SVM’s many appealing features, generalization ability, that is the ability to accurately predict outcome values for previously unseen data, can help minimize cross-unit prediction errors. The basic principle of SVM is to construct the maximum margin hyperplanes to separate data points into different classes. Maximizing the margin reduces complexity of the classification function, thus minimizing the possibility of overfitting. Therefore, better generalization can be achieved intrinsically for SVM [38]. When many classes are involved, like the polymer classification example in this study, the hier-SVM algorithm was shown to be beneficial, because this multilevel classification scheme facilitates refined classification for chemically similar materials to achieve more accurate prediction [26]. In addition, the TreeBagger algorithm is based on random forest, which is one of the most powerful classifiers in machine learning [39]. However, for the current study, the cross-unit performance of TreeBagger was not as good as the SVM algorithms. 

The combination of the hardware design and implementation of advanced calibration techniques results in a repeatable and reproducible performance between different MicroNIR™ spectrometers, allowing effective direct model transferability. However, it is not intended to say that this will be the ultimate solution that eliminates all problems that necessitate calibration transfer. The scope of the current study was limited to model transferability only involving instrument-to-instrument differences, not very heterogeneous samples, and data collected with sound sampling and measurement protocols. For example, when different instruments are placed in different environments, environmental changes may have to be corrected for the model via calibration transfer. Very heterogeneous samples, such as biological samples, will be more difficult to handle in general. Even very small instrument-to-instrument differences could cause unsatisfactory cross-unit prediction results. A global model approach using data from samples with all expected sources of variance and/or measured with multiple instruments for calibration could significantly minimize prediction errors. Model updating techniques will also be very helpful [40]. Direct model transferability will be evaluated for very heterogeneous materials in our future studies. In addition, poor cross-unit model performance often results from nonqualified calibration data that are not collected with a careful sampling plan and a proper measurement protocol. The success of a multi-instrument NIR project must start with reliable NIR data that are collected with best practices in sampling [41,42] and measurement [43,44].

The current study demonstrated the possibility of direct model transfer from instrument to instrument for both classification and quantification problems, which has laid a good foundation for the use of a large number of compact NIR instruments. More studies should be encouraged in wider applications and using all kinds of instruments from various manufacturers. Scalability of handheld and process NIR solutions can become more manageable when the number of times that calibration transfer has to be performed between instruments can be minimized. 

## 4. Materials and Methods

### 4.1. Materials

For the polymer classification study, 46 injection molded resins were obtained from The ResinKit™ (The Plastics Group of America, Woonsocket, RI, USA). The set of resins contains a variety of polymer materials, as well as various properties within the same type of material (for example different densities or strengths). Each resin was treated as an individual class in this study. All the resins used in this study are listed in Table 6 and detailed properties of these materials are available upon request. To evaluate the cross-kit prediction performance, three resin kits were used.

For the API quantification study, 48 pharmaceutical powders consisting of different concentrations of three crystalline active ingredients, as well as two amorphous excipients were provided by Prof. Heinz W. Siesler at University of Duisburg-Essen, Germany [4]. The active ingredients used were acetylsalicylic acid (ASA, Sigma-Aldrich Chemie GmbH, Steinheim, Germany), ascorbic acid (ASC, Acros Organics, NJ, USA), and caffeine (CAF, Sigma-Aldrich Chemie GmbH, Steinheim, Germany), and the two excipients used were cellulose (CE, Fluka Chemie GmbH, Buchs, Switzerland) and starch (ST, Carl Roth GmbH, Karlsruhe, Germany). The concentration of the active ingredients ranged from 13.77–26.43% (*w*/*w*), and all samples consisted of 40% (*w*/*w*) of a 3:1 (*w*/*w*) mixture of cellulose and starch.

### 4.2. Spectra Collection

#### 4.2.1. Resin Samples

Three MicroNIR™ OnSite spectrometers (Viavi Solutions Inc., Santa Rosa, CA, USA) in the range of 908–1676 nm were randomly picked to collect the spectra of the resin samples. The spectral bandwidth is ~1.1% of a given wavelength. Three kits of samples were measured in the diffuse reflection mode. A MicroNIR™ windowless collar was used to interface with the samples, which optimized the sample placement relative to the spectrometer. Each sample was placed between the windowless collar of the MicroNIR™ spectrometer and a 99% diffuse reflection standard (Spectralon^®^, LabSphere, North Sutton, NH, USA). The reason for using the Spectralon^®^ behind each sample was to return signal back to the spectrometer, particularly for very transparent samples, in order to improve the signal-to-noise ratio.

Each sample was scanned in five specified locations to account for the most variation in sample shape and thickness. In addition, at each position the sample was scanned in two orientations with respect to the MicroNIR™ lamps to account for any directionality in the structure of the molding. For each position and orientation, three replicate scans were acquired, totaling thirty scans per sample, per spectrometer. The MicroNIR™ spectrometer was re-baselined after every ten samples, using a 99% diffuse reflectance reference scan (Spectralon^®^), as well as a lamps-on dark scan, in which nothing was placed in front of the spectrometer. Each sample was measured by all three spectrometers following the same protocol.

#### 4.2.2. Pharmaceutical Samples

Each of the 48 samples were placed in individual glass vials, and their spectra were collected by three randomly picked MicroNIR™ 1700ES spectrometers in the range of 908–1676 nm using the MicroNIR™ vial-holder accessory. The spectral bandwidth is ~1.1% of a given wavelength. In this measurement setup, the samples were scanned from the bottom of the vial in the diffuse reflection mode.

Each sample was scanned twenty times using each MicroNIR™ spectrometer. The sample was rotated in the vial-holder between every scan to account for sample placement variation, as well as the non-uniform thickness of the vial. Before every new sample, the MicroNIR™ spectrometer was re-baselined by scanning a 99% diffuse reflectance reference (Spectralon^®^), as well as a lamps-on dark scan, which consisted of an empty vial in place of a sample. Each sample was measured by all three spectrometers following the same protocol.

### 4.3. Data Processing and Multivariate Analysis

#### 4.3.1. Polymer Classification

All steps of spectral processing and chemometric analysis were performed using MATLAB (The MathWorks, Inc., Natick, MA). All spectra collected were pretreated using Savitzky-Golay first derivative followed by standard normal variate (SNV).

PLS-DA, SIMCA, TreeBagger, SVM and hier-SVM were applied to preprocessed datasets. Autoscaling was performed when running these algorithms. To implement PLS-DA, the number of PLS factors was chosen by training set cross validation and the same number was used for all classes. To implement SIMCA, the number of principal components (PC) was optimized for each class by training set cross validation. No optimization was performed for TreeBagger, SVM and hier-SVM, and the default settings were used. For TreeBagger, the number of decision trees in the ensemble was set to be 50. Since random selection of sample subsets and variables is involved when running TreeBagger, there are small differences in the results from run to run. To avoid impacts from these differences, all the TreeBagger results were based on the mean of 10 runs. For SVM algorithms, the linear kernel with parameter C of 1 was used.

For the same-unit-same-kit performance, the models built with data collected from four locations on each sample in one resin kit by one spectrometer were used to predict data collected from the other location on each sample in the same resin kit by the same spectrometer. For the same-unit-cross-kit performance, the models built with all the data collected from one resin kit by one spectrometer were used to predict all the data collected from a different resin kit by the same spectrometer. For the cross-unit-same-kit performance, the models built with all the data collected from one resin kit by one spectrometer were used to predict all the data collected from the same resin kit by a different spectrometer. For the cross-unit-cross-kit performance, the models built with all the data collected from one resin kit by one spectrometer were used to predict all the data collected from a different resin kit by a different spectrometer. 

#### 4.3.2. API Quantification

All steps of spectral processing and chemometric analysis were performed using MATLAB. Some functions in PLS_Toolbox (Eigenvector Research, Manson, WA, USA) were called in the MATLAB code. To develop the calibration models, 38 out of the 48 samples were selected as the calibration samples via the Kennard-Stone algorithm based on the respective API concentration. The remaining 10 samples were used as the validation samples. The preprocessing procedure was optimized for each API separately based on the calibration set cross validation. The same preprocessing procedure was used on all three instruments for the same API. PLS models were developed using the corresponding preprocessed datasets for each API.

To evaluate the same-unit performance, the model built on one instrument was used to predict the validation set collected by the same instrument. To evaluate the cross-unit performance without calibration transfer, the model built on one instrument was used to predict the validation set collected by the other instruments. 

For calibration transfer demonstration, Unit 1 was used as the master instrument, and Unit 2 and Unit 3 were used as the slave instruments. Eight transfer samples were selected from the calibration samples with the Kennard-Stone algorithm. To perform bias correction, bias was determined using the transfer data collected by the slave instrument, and the bias was applied to the predicted values using the validation data collected by the slave instrument. To perform PDS, the window size was optimized based on RMSEP, and the corresponding lowest RMSEP was reported in this study. To perform GLS, parameter *a* was optimized based on RMSEP, and the corresponding lowest RMSEP was reported in this study.

## 5. Conclusions

In this study, direct model transferability was investigated when multiple MicroNIR™ spectrometers were used. As demonstrated by the polymer classification example, high prediction success rates can be achieved for the most stringent cross-unit-cross-kit cases with multiple algorithms including the widely used SIMCA method. Better performance was achieved with SVM algorithms, especially when a hierarchical approach was used (hier-SVM). As demonstrated by the API quantification example, low prediction errors were achieved for the cross-unit cases with PLS models. These results indicate that the direct use of a model developed on one MicroNIR™ spectrometer on the other MicroNIR™ spectrometers is possible. The successful direct model transfer is enabled by the robust design of the MicroNIR™ hardware and will make deployment of multiple spectrometers for various applications more manageable and economical.

## Figures and Tables

**Table 1:** Prediction success rates (%) of polymer classification.

Algorithm	Unit# Kit# for Modeling	Unit# Kit# for Testing					
		Unit1 K1	Unit2 K1	Unit3 K1	Unit1 K2	Unit2 K2	Unit3 K3
PLS-DA	Unit 1 K1	99.64	89.68	83.99	95.87	88.91	82.39
	Unit 2 K1	91.96	100	81.52	90.87	99.57	84.49
	Unit 3 K1	76.74	75.32	100	75.07	73.12	99.20
SIMCA	Unit 1 K1	100	99.42	96.45	99.35	97.32	96.81
	Unit 2 K1	98.77	100	95.43	97.68	99.93	95.80
	Unit 3 K1	96.30	93.29	100	96.09	92.17	100
TreeBagger	Unit 1 K1	100	97.11	95.80	98.04	95.94	96.30
	Unit 2 K1	97.83	100	93.55	94.49	98.26	96.16
	Unit 3 K1	95.14	98.41	100	96.09	98.84	98.84
SVM	Unit 1 K1	100	99.86	97.54	98.26	97.90	97.83
	Unit 2 K1	98.70	100	97.03	94.93	98.26	98.26
	Unit 3 K1	97.83	96.18	100	96.30	95.00	99.57
Hier-SVM	Unit 1 K1	100	100	97.97	97.83	97.83	97.25
	Unit 2 K1	99.93	100	98.26	98.26	99.13	99.13
	Unit 3 K1	99.13	100	100	96.88	97.83	100

**Table 2:** Number of missed predictions of polymer classification in the format of number of missed predictions/total number of predictions.

Algorithm	Unit# Kit# for Modeling	Unit# Kit# for Testing					
		Unit1 K1	Unit2 K1	Unit3 K1	Unit1 K2	Unit2 K2	Unit3 K3
PLS-DA	Unit 1 K1	1/276	143/1386	221/1380	57/1380	153/1380	243/1380
	Unit 2 K1	111/1380	0/277	255/1380	126/1380	6/1380	214/1380
	Unit 3 K1	321/1380	342/1386	0/276	344/1380	371/1380	11/1380
SIMCA	Unit 1 K1	0/276	8/1386	49/1380	9/1380	37/1380	44/1380
	Unit 2 K1	17/1380	0/277	63/1380	32/1380	1/1380	58/1380
	Unit 3 K1	51/1380	93/1386	0/276	54/1380	108/1380	0/1380
TreeBagger	Unit 1 K1	0/276	40/1386	58/1380	27/1380	56/1380	51/1380
	Unit 2 K1	30/1380	0/277	89/1380	76/1380	24/1380	53/1380
	Unit 3 K1	67/1380	22/1386	0/276	54/1380	16/1380	16/1380
SVM	Unit 1 K1	0/276	2/1386	34/1380	24/1380	29/1380	30/1380
	Unit 2 K1	18/1380	0/277	41/1380	70/1380	24/1380	24/1380
	Unit 3 K1	30/1380	53/1386	0/276	51/1380	69/1380	6/1380
Hier-SVM	Unit 1 K1	0/276	0/1386	28/1380	30/1380	30/1380	38/1380
	Unit 2 K1	1/1380	0/277	24/1380	24/1380	12/1380	12/1380
	Unit 3 K1	12/1380	0/1386	0/276	43/1380	30/1380	0/1380

**Table 3:** The normalized root mean square error of prediction (NRMSEP, %) for ASA.

Test Sets	No Correction			Bias	PDS	GLS
	Unit 1	Unit 2	Unit 3	Unit 1	Unit 1	Unit 1
Unit 1	3.4	3.5	3.5	-	-	-
Unit 2	4.0	4.2	3.9	3.7	3.3	3.6
Unit 3	4.3	4.5	4.2	4.1	3.5	4.4

**Table 4:** The normalized root mean square error of prediction (NRMSEP, %) for ASC.

Test Sets	No Correction			Bias	PDS	GLS
	Unit 1	Unit 2	Unit 3	Unit 1	Unit 1	Unit 1
Unit 1	3.0	2.6	2.7	-	-	-
Unit 2	2.7	2.7	2.6	2.3	3.5	2.6
Unit 3	2.5	2.5	2.7	2.2	3.1	2.4

**Table 5:** The normalized root mean square error of prediction (NRMSEP, %) for CAF.

Test Sets	No Correction			Bias	PDS	GLS
	Unit 1	Unit 2	Unit 3	Unit 1	Unit 1	Unit 1
Unit 1	4.0	4.6	3.7	-	-	-
Unit 2	4.1	4.7	4.2	4.2	4.3	3.2
Unit 3	4.2	4.9	4.0	4.1	6.2	3.9

**Table 6:** Polymer materials used for the classification study.

No.	Polymer Type	No.	Polymer Type
1	PolyStyrene-General Purpose	24	Polyethylene-High Density
2	PolyStyrene-High Impact	25	Polypropylene-Copolymer
3	Styrene-Acrylonitrile (SAN)	26	Polypropylene-Homopolymer
4	ABS-Transparent	27	Polyaryl-Ether
5	ABS-Medium Impact	28	Polyvinyl Chloride-Flexible
6	ABS-High Impact	29	Polyvinyl Chloride-Rigid
7	Styrene Butadiene	30	Acetal Resin-Homopolymer
8	Acrylic	31	Acetal Resin-Copolymer
9	Modified Acrylic	32	Polyphenylene Sulfide
10	Cellulose Acetate	33	Ethylene Vinyl Acetate
11	Cellulose Acetate Butyrate	34	Urethane Elastomer (Polyether)
12	Cellulose Acetate Propionate	35	Polypropylene-Flame Retardant
13	Nylon-Transparent	36	Polyester Elastomer
14	Nylon-Type 66	37	ABS-Flame Retardant
15	Nylon-Type 6 (Homopolymer)	38	Polyallomer
16	Thermoplastic Polyester (PBT)	39	Styrenic Terpolymer
17	Thermoplastic Polyester (PETG)	40	Polymethyl Pentene
18	Phenylene Oxide	41	Talc-Reinforced Polypropylene
19	Polycarbonate	42	Calcium Carbonate-Reinforced Polypropylene
20	Polysulfone	43	Nylon (Type 66–33% Glass)
21	Polybutylene	44	Thermoplastic Rubber
22	Ionomer	45	Polyethylene (Medium Density)
23	Polyethylene-Low Density	46	ABS-Nylon Alloy

## Supplementary Materials

The supplementary materials on reproducibility of MicroNIR™ products are available online https://www.mdpi.com/1420-3049/24/10/1997/s1. Figure S1: MicroNIR™ OnSite manufacturing data demonstrating instrument-to-instrument reproducibility, Figure S2: MicroNIR™ 1700ES manufacturing data demonstrating instrument-to-instrument reproducibility.
