# Supplementary material for: Investigation of Direct Model Transferability Using Miniature Near-Infrared Spectrometers
Source: Molecules. 2019 May 24;24(10):1997. doi: 10.3390/molecules24101997 (PMC6571657; doi:10.3390/molecules24101997)
Supplement: Supplementary file 1 [file molecules-24-01997-s001.pdf]

Supplementary Materials – Reproducibility of MicroNIR™ Products

The robust design of the MicroNIR™ hardware makes it possible to manufacture instruments with high instrument-to-instrument reproducibility and the on-board calibrations enable accurate and repeatable performances between instruments. Each MicroNIR™ is subject to stringent performance testing and characterization during product manufacturing to ensure compliance with U.S. Pharmacopoeia spectroscopic performance standards (USP1119). Figure S1 displays manufacturing data from the three MicroNIR™ OnSite units used for the polymer classification study, and Figure S2 displays manufacturing data from the three MicroNIR™ 1700ES units used for the API regression study.

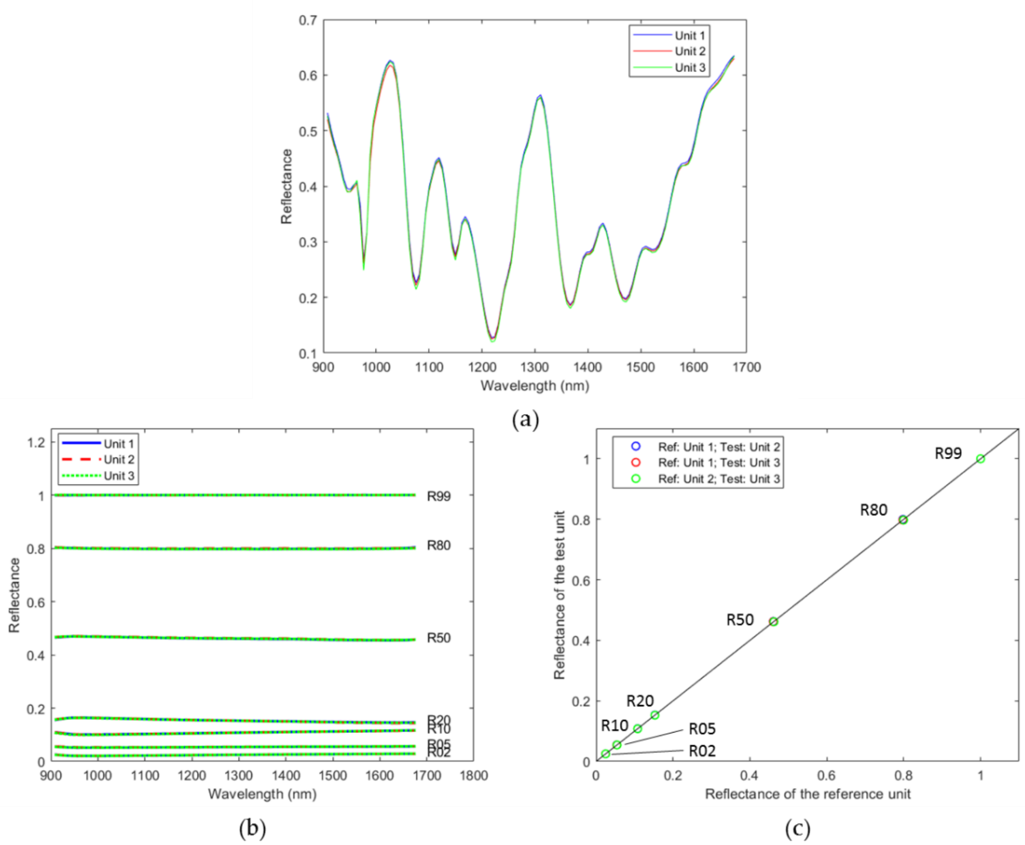

Figure S1. MicroNIR™ OnSite manufacturing data demonstrating instrument-to-instrument reproducibility. (**a**) Scans of Avian's WCR-2066 wavelength standard; (**b**) Scans of Avian's Fluorilon Gray Scale Standards; (**c**) Photometric Linearity between different instruments.

Figure S1a and Figure S2a display the reflectance measurements of Avian's WCR-2066 reflective wavelength reference standard (Avian Technologies LLC, NH, USA), equivalent to NIST's SRM-2036, collected during the manufacturing of the three MicroNIR™ OnSite and three MicroNIR™ 1700ES units used in these studies, respectively. This standard consists of rare earth oxide glass in physical contact with a piece of sintered PTFE. This standard has well-defined absorption peaks and can be used to validate the MicroNIR™ wavelength calibration.

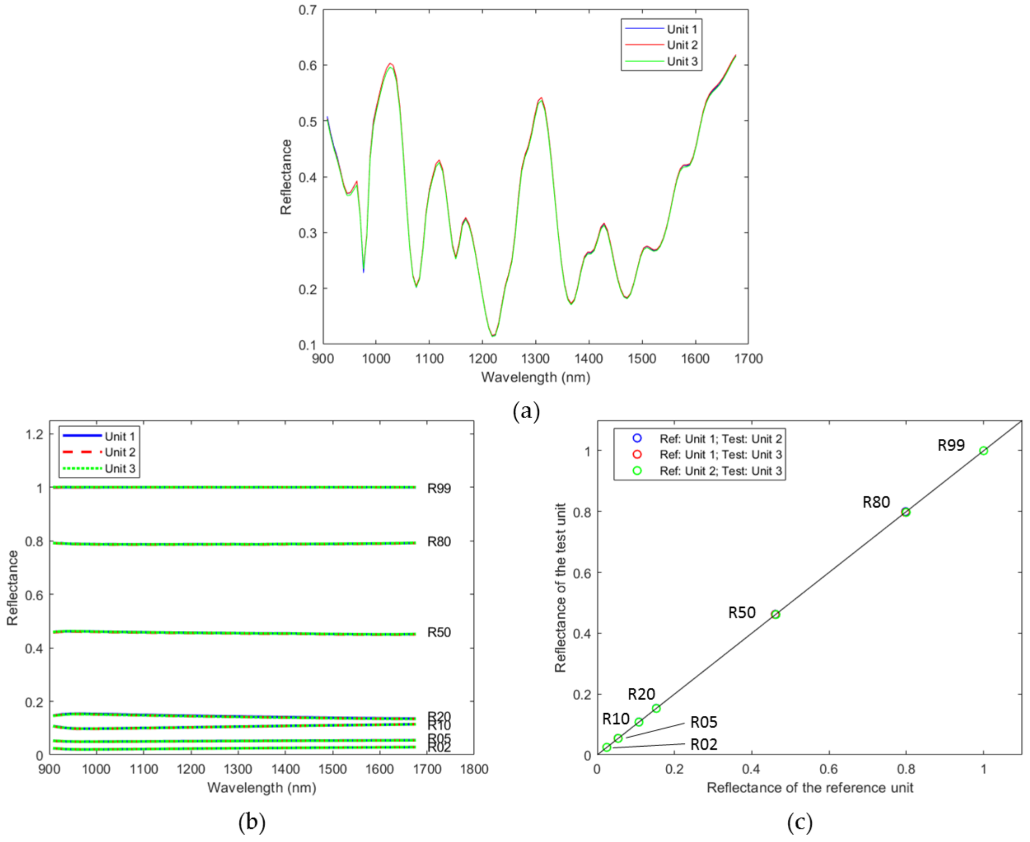

Figure S2. MicroNIR™ 1700ES manufacturing data demonstrating instrument-to-instrument reproducibility. (**a**) Scans of Avian's WCR-2066 wavelength standard; (**b**) Scans of Avian's Fluorilon Gray Scale Standards; (**c**) Photometric Linearity between different instruments.

Figure S1b and Figure S2b display the photometric responses of gray-scale diffuse reflectance standards (Fluorilon Gray Scale Standards, Avian Technologies LLC, NH, USA) collected during the manufacturing of the three MicroNIR™ OnSite and three MicroNIR™ 1700ES units used in these studies, respectively. These reflectance standards consist of different levels of carbon-doped sintered PTFE, resulting in a relatively flat spectral response across the MicroNIR wavelength range. The amount of carbon concentration in each sample determines the level of reflectance, which allows for measuring over most of the dynamic range.

Figure S1c and Figure S2c demonstrate the photometric linearity between instruments using the gray-scale diffuse reflectance measurements of Figure S1b and Figure S2b, respectively. The responses of reference units are plotted against the responses of the test units for a single wavelength. For demonstration purposes, the three units were tested against each other and a central wavelength of 1298.3 nm was used, though these results are typical for the entire operating wavelength range of the MicroNIR™ instruments. The black line in these two figures define a slope equal to one and a y-intercept equal to zero, which agrees well with the data points indicating similar photometric responses between instruments.
